# Surrogate Markers of Intestinal Permeability, Bacterial Translocation and Gut–Vascular Barrier Damage Across Stages of Cirrhosis

**DOI:** 10.1111/liv.70119

**Published:** 2025-05-02

**Authors:** Frederic Haedge, Philipp A. Reuken, Johanna Reißing, Karsten Große, Mick Frissen, Majda El‐Hassani, Rene Aschenbach, Ulf Teichgräber, Andreas Stallmach, Tony Bruns

**Affiliations:** ^1^ Department of Internal Medicine III University Hospital RWTH Aachen Aachen Germany; ^2^ Department of Internal Medicine IV Jena University Hospital, Friedrich Schiller University Jena Jena Germany; ^3^ Department of Radiology Jena University Hospital, Friedrich Schiller University Jena Jena Germany

**Keywords:** acute‐on‐chronic liver failure, gut–barrier dysfunction, gut–liver axis, intestinal permeability, leaky gut, liver cirrhosis

## Abstract

**Background and Aims:**

Portal hypertension, gut barrier dysfunction, and pathological bacterial translocation are hallmarks of cirrhosis driving complications. As measuring gut barrier function is demanding, surrogate markers have been proposed, but their intercorrelation and applicability across different stages of advanced liver disease, particularly in acute‐on‐chronic liver failure (ACLF), are largely unknown.

**Methods:**

Proposed markers of gut barrier dysfunction and bacterial translocation were quantified in sera from 160 patients with cirrhosis across different disease stages of compensated and decompensated cirrhosis as well as from 20 patients in hepatic and portal vein serum before and after the insertion of transjugular intrahepatic portosystemic stent (TIPS) using enzyme‐linked immunosorbent assay (ELISA).

**Results:**

Across all stages of liver disease, the gut–vascular barrier (GVB) marker plasmalemma vesicle protein‐1 (PV‐1) correlated with bacterial translocation markers endogenous endotoxin‐core IgA antibodies (EndoCAb) and LPS‐binding protein (LBP) but not with intestinal damage markers intestinal fatty acid binding protein (I‐FABP) and zonulin‐family peptides (ZFP). PV‐1 and EndoCAb were higher in decompensated cirrhosis without further increase in ACLF. Among investigated markers, only I‐FABP correlated with the portosystemic pressure gradient, and TIPS insertion significantly reduced portal concentrations within 24 h. Higher PV‐1 levels indicated poor transplant‐free survival in univariate and multivariable analysis.

**Conclusions:**

Surrogate markers of bacterial gut barrier dysfunction and bacterial translocation like ZFP, LBP and EndoCAb appear of limited use in advanced stages of cirrhosis and are confounded by hepatic synthesis capacity, portal congestion and acute phase responses. The prognostic implications of circulating PV‐1 in decompensated cirrhosis levels demand further investigation.

**Summary**

- Gut barriers are comprised of multiple layers, including mucus, the epithelium, a network of mononuclear phagocytes, and cells of the endothelium, which form a gut–vascular barrier (GVB) to the circulatory system. In patients with cirrhosis, gut dysbiosis, increased intestinal permeability (IP), and an impaired immune system lead to a ‘leaky gut’.
- Different markers found in blood samples, such as zonulin‐family peptides (ZFP) and I‐FABP for gut permeability, EndoCAb and LBP for bacterial translocation, and PV‐1 for gut‐vascular barrier damage, are used to assess gut barrier function in cirrhosis.
- Our study shows that these markers do not correlate well in advanced stages of cirrhosis, specifically in acute‐on‐chronic liver failure (ACLF).
- Notably, I‐FABP levels are related to portal pressure and decrease rapidly after reducing this pressure.
- PV‐1 is often found in the blood of patients with decompensated cirrhosis, is linked to markers of bacterial translocation, and indicates poor survival.

## 1 Introduction

The intestinal barrier is a complex, multilayered structure consisting of physical, chemical, microbial and immunological components. The mucus layer, produced and maintained by goblet cells, serves as the primary physical barrier between the microbiota and the epithelial cells. The epithelium itself is a single layer of columnar epithelial cells connected by tight junctions. Beneath this layer, the body's largest system of mononuclear phagocytes, including intestinal macrophages and dendritic cells (DCs), functions to engulf invading microorganisms and luminal materials. Finally, endothelial cells form the gut–vascular barrier (GVB), separating the gut from the circulatory system [1]. This barrier is not impermeable, allowing bacteria and their toxins to migrate from the intestines to the lymph nodes, liver, spleen, or bloodstream—a process known as bacterial translocation [2].

The prevalence of BT to MLN is reported to range between 4% and 59%, with the highest rates observed in patients with intestinal obstruction or Crohn's disease [3, 4]. In patients without bowel obstruction or inflammatory bowel disease, the translocation rate is approximately 5%, which may represent the physiological rate of translocation in humans [5]. Pathological BT has been described in a range of intestinal and extraintestinal diseases, including intestinal obstruction [3], celiac disease [6], Crohn's disease [4], cirrhosis [7] and depression [8]. In patients with cirrhosis, three involved principal mechanisms promoting pathological BT include intestinal dysbiosis [9], increased intestinal permeability (IP) [10] and impaired immune responses [11]. In addition, multiple factors like malnutrition [12], genetic predisposition [13] and changes in bile acids [14, 15] are known to modulate intestinal barrier function and BT.

Assessing IP in cirrhosis may help identify patients at risk for complications in clinical practise [16]. However, gold standards for assessing IP are invasive, such as the lactulose‐mannitol test [17, 18, 19] or (51)Cr‐ethylenediaminetetraacetic acid‐labelled EDTA [16], complex, and time‐consuming, and therefore rarely done outside of clinical studies. In patients without liver disease, several blood or stool surrogate markers of gut barrier function have been proposed to diagnose intestinal barrier dysfunction and its consequences, including (1) endotoxemia markers like endogenous endotoxin‐core antibody (EndoCAb) and the acute phase protein Lipopolysaccharide binding protein (LBP) [20, 21], (2) intestinal damage markers like zonulin‐family peptides (ZFPs) and intestinal fatty acid binding protein (I‐FABP) also known as fatty acid binding protein 2 [22, 23, 24, 25], or (3) the plasmalemma vesicle protein‐1 (PV‐1), an endothelial cell‐specific glycoprotein, that was shown to be essential for the regulation of endothelial homeostasis and permeability of the GVB [26, 27].

In general, an ideal biomarker should combine several properties: (1) they should be non‐invasive, easy to measure, and inexpensive; (2) they should be able to be collected from easily accessible sources such as blood or urine; (3) they should have a high sensitivity that enables early detection and no overlap of values between diseased patients and healthy controls; (4) they should have a high specificity, that is, they should be strongly upregulated (or downregulated) especially in the diseased samples and not be influenced by concomitant diseases; and (5) their values should help in the stratification of risk and have a prognostic power in relation to outcomes [28]. While the aforementioned surrogate markers of gut barrier dysfunction and BT fulfil these criteria, already only limited in diseases like inflammatory bowel disease, irritable bowel syndrome, or celiac disease [29, 30, 31, 32], their regulation in advanced liver disease, particularly in cirrhosis, acute decompensation, and acute‐on‐chronic liver failure (ACLF) is largely unknown.

Therefore, the aim of this study was to comparatively investigate surrogate markers of IP, bacterial translocation and GVB damage across different stages of cirrhosis and to assess portal vein concentrations and immediate effects of portal decompression after transjugular intrahepatic stent shunt (TIPS) insertion in patients with recurrent ascites.

## 2 Patients and Methods

### 2.1 Patients

This is a bicenter retrospective analysis comprising analyses using frozen serum samples from three cohorts of patients: (i) 120 patients with decompensated cirrhosis and ascites who underwent diagnostic ascitic tap or therapeutic paracentesis, (ii) 40 age‐matched and gender‐matched patients with compensated cirrhosis and (iii) 20 patients who underwent TIPS insertion for recurrent or refractory ascites. Patients with decompensated cirrhosis were admitted to the Jena University Hospital or the University Hospital RWTH Aachen due to acute decompensation with underlying liver cirrhosis with newly occurring ascites grade 2 and 3 or worsening ascites, while patients presenting as outpatients for routine large volume paracentesis for refractory ascites were not included in the study. Patients with peritoneal carcinomatosis or secondary peritonitis were excluded. The samples and clinical data of the patients were collected at study inclusion, which was defined as the time of paracentesis. Patients were prospectively followed for the occurrence of death from any cause or liver transplantation within 90 days. Patients were stratified for the presence of ACLF according to the Chronic Liver Failure Consortium (CLIF) criteria [33].

Sera from age‐matched and gender‐matched patients with compensated cirrhosis were selected using frozen biomaterial from patients with compensated cirrhosis recruited for a previous genetic association study [34]. Paired serum samples from the portal vein (PV) and the right or middle hepatic vein (HV) at 2 days were collected from 20 patients with decompensated cirrhosis who underwent implantation of a transjugular intrahepatic portosystemic shunt (TIPS) for recurrent or refractory ascites at the Jena University Hospital of Jena between 2018 and 2022.

The study was approved by the local ethics committees (Ethikkommission der Friedrich‐Schiller Universität Jena an der Medizinischen Fakultät: 3150‐06/11, 2880‐08/10, 2018‐1080_2‐BO and Ethik‐Kommission an der Medizinischen Fakultät der RWTH Aachen: EK 327/19). Written informed consent was obtained from the patients before study inclusion. Serum centrifugation was performed as described previously [35], and sera were stored at −80°C until analysis. At the beginning of the study, demographic and clinical data, including the aetiology of liver cirrhosis, Child‐Pugh and the model for end‐stage liver disease (MELD) score, were documented. Clinical chemistry parameters were determined by routine laboratory tests.

### 2.2 Enzyme‐Linked Immunosorbent Assay

PV‐1 was measured in human serum samples by enzyme‐linked immunosorbent assays (ELISA) (Immunosorbent Assay Kit; Biomatik) following the manufacturer's instructions. ZFPs were measured in human serum samples by ELISA assay (Immundiagnostik AG, Bensheim, Germany), EndoCAb IgA, LBP and I‐FABP (HycultBiotech) by enzyme‐linked‐immunosorbent assays according to the manufacturer's instructions. The limits of detection of the ELISAs as communicated by the manufacturers were < 0.062 ng/mL, 47 pg/mL, 0.183 ng/mL, 0.16 AMU/mL and 4.4 ng/mL for PV‐1, I‐FABP, ZFP, EndoCAb IgA and LBP, respectively. Serum samples were used undiluted (PV‐1) or in dilutions of 1:5 (I‐FABP), 1:20 (ZFP), 1:100 (EndoCAb IgA), or 1:2500 (LBP).

### 2.3 Transjugular Intrahepatic Portosystemic Stent

TIPS (8–10 mm Viator, W.L. Gore, Newark, Delaware, USA) placement was conducted following local standard operating procedures. Invasive measurement of portal and vena cava inferior pressures was performed using a pressure transducer system (Fa Braun, Melsungen, Germany; Aachen). The portal pressure gradient (PPG) was determined as the difference between portal and vena cava inferior pressures. PPG measurements were obtained immediately after TIPS placement. Hepatic venous samples were collected from the respective HV selected for TIPS placement prior to the puncture of the PV. Blood samples from the PV were collected immediately after PV puncture, prior to tract dilation or TIPS insertion. Immediately after collecting whole blood from the PV and HV, samples were centrifuged at 2000 g for 10 min. Serum samples were then stored at −80°C until further use.

### 2.4 Statistical Analysis

The statistical analyses and data presentation were performed using IBM SPSS (version 29), StataNow 18.5 MP—Parallel Edition (StataCorp LLC, TX, USA) and Prism version 10.01 (GraphPad, San Diego, CA, USA). To compare two groups, we used Mann–Whitney U tests for continuous variables and Fisher's exact test for nominal variables. Continuous variables from paired samples were compared using the Wilcoxon signed‐rank test. Bivariate nonparametric correlation analysis (Spearman's rho, rs) was performed to identify correlations between continuous or ordinal variables. Time‐to‐event analyses were performed using Kaplan–Meier methods, with groups being contrasted by log‐rank tests. Continuous variables were dichotomised according to the maximum Youden index in receiver operating characteristics (ROC) analysis. Univariate and multivariable analysis of risk factors for mortality were assessed by Cox regression. Competing risk analysis of 90‐day mortality was performed using the Fine–Grey subdistribution hazard model, treating liver transplantation as a competing event.

## 3 Results

### 3.1 Patients Characteristics

The groups of 120 patients with decompensated cirrhosis and ascites and of 40 patients with compensated Child‐Pugh A cirrhosis were matched for sex and age and well balanced for platelet count and alanine transaminase levels (Table 1). Notably, there was a higher number of patients with alcohol‐related liver disease (ALD) in decompensated patients. As expected, patients with decompensated cirrhosis had more advanced liver disease with higher levels of Child–Pugh stage, MELD score, and signs of systemic inflammation including white blood cell count (WBC) and CRP levels (Table 1).

**TABLE 1:** Baseline characteristics of patients with cirrhosis.

	Compensated cirrhosis (*n* = 40)	Decompensated cirrhosis (*n* = 120)	*p*
Age (years)	64 (56–71)	61 (53–68)	0.20
Male sex	18 (45%)	75 (63%)	0.06
Aetiology			
ALD	10 (25%)	95 (79%)	< 0.0001
Viral	9 (23%)	8 (7%)	
Other	21 (53%)	17 (14%)	
ALCF			
No ACLF (AD)	40 (100%)	78 (65%)	N/A
ACLF Grade 1		21 (17.5%)	
ACLF Grade 2		13 (10.83%)	
ACLF Grade 3		8 (6.67%)	
Child‐Pugh stage			
A	40 (100%)		N/A
B		30 (25%)	
C		90 (75%)	
Previous decompensation	0	73 (61%)	
Previous ascites		64 (88%)^a^	
Previous bleeding		13 (18%)^a^	
Previous hepatic encephalopathy		14 (19%)^a^	
HCC	10 (25%)	25 (21%)	0.66
MELD score	7 (6–9)	19 (13–23)	< 0.0001
Total serum bilirubin (mg/dL)	0.8 (0.6–1.1)	2.7 (1.5–7.1)	< 0.0001
INR	1.05 (1–1.1)	1.5 (1.3–1.8)	< 0.0001
Creatinine (mg/dL)	0.8 (0.7–1.0)	1.2 (0.7–1.7)	0.0007
Albumin (g/L)	36 (32–38)	25 (20–29)	< 0.0001
Sodium (mmol/L)	138 (137–140)	135 (132–138)	< 0.0001
WBC (/nL)	5.5 (4.7–7.75)	7.45 (5.1–10.5)	0.008
Platelets (/nL)	157 (93–200)	120 (75–182)	0.06
ALT (U/L)	44 (33–72)	36 (25–70)	0.08
AST (U/L)	46 (30–70)	68 (42–136)	0.0004
CRP (mg/L)	3 (2–7)	32 (15–63)	< 0.0001
NSBB use	10 (25%)	61 (51%)	0.006

*Note:* Data are given as number with fractions or medians with interquartiles. Mann–Whitney U test for continuous and Fisher's exact test for categorial variables.

Abbreviations: ACLF, acute‐on‐chronic liver failure; ALD, alcohol‐related liver disease; ALT, alanine aminotransferase; AST, aspartate aminotransferase; CRP, C‐reactive protein; HCC, hepatocellular carcinoma; MELD, model for end‐stage liver disease; N/A, not applicable; NSBB, non‐selective beta blockers; WBC, white blood cell count.

^a^ Sum exceeds 100%.

### 3.2 Gut Permeability and Translocation Markers in Decompensated Cirrhosis and ACLF

Based on the severity and trajectory of decompensated cirrhosis, patients were stratified into 3 groups: 78 patients with acute decompensation (AD), 21 patients with grade 1 ACLF, and 21 patients with ACLF grade 2 or higher. The organ failure profiles are described in the Table S1 [36].

When comparing the patients in the compensated stage of cirrhosis with patients with cirrhosis and ascites, serum levels of PV‐1 (median 0.016 vs. 0.166 ng/mL; *p* < 0.0001) and EndoCAb (median 56.23 vs. 147.2 AMU/mL; *p* = 0.0025) were higher in patients with decompensated cirrhosis than in patients with compensated liver disease, while serum levels of ZFP were lower (median 18.28 vs. 6.72 ng/mL; *p* < 0.0001). There were no significant differences between these two groups in terms of circulating LBP (median 18.99 vs. 19.98 μg/mL; *p* = 0.8486) or I‐FABP concentrations (median 0.716 vs. 0.4830 ng/mL; *p* = 0.854).

**FIGURE 1:**
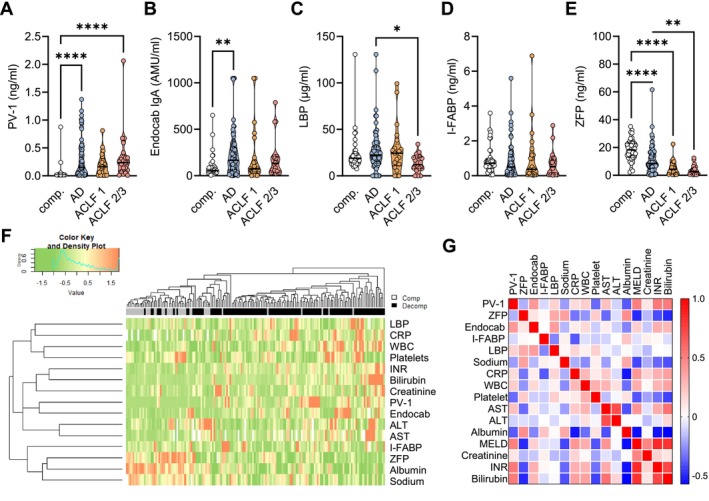
Peripheral serum concentrations of surrogate markers of intestinal permeability, bacterial translocation, and gut–vascular barrier damage across different stages of cirrhosis. (A–E) Serum concentrations of circulating (A) PV‐1, (B) EndoCAb IgA, (C) LBP, (D) I‐FABP and (E) ZFP from patients with compensated cirrhosis (*n* = 40), acutely decompensated cirrhosis without ACLF (AD, *n* = 78), acute‐on‐chronic liver failure grade 1 (ACLF *n* = 21) and ACLF grade 2 or higher (*n* = 21). Truncated violin plots with overlaying individual values are shown. (F) Hierarchical cluster analysis of individual serum concentrations and routine laboratory values. (G) Correlation matrix showing non‐parametric correlation coefficients of the different markers with routine laboratory parameters and the MELD score. *p < 0.05; **p < 0.01; ****p < 0.0001 in Kruskal–Wallis test with Dunn's post hoc test. AD, acute decompensation; ACLF, acute‐on‐chronic liver failure; ALT, alanine aminotransferase; AST, aspartate aminotransferase; comp., compensated; CRP, C‐reactive protein; EndoCAb, endotoxemia markers like endogenous endotoxin‐core antibody; I‐FABP, intestinal fatty acid binding protein; LBP, Lipopolysaccharide binding protein; MELD, model for end‐stage liver disease; PV‐1, plasmalemma vesicle protein‐1; WBC, white blood cell count; ZFP, Zonulin‐family peptides.

When patients with decompensated cirrhosis were stratified for the presence and severity of ACLF, we did observe a strong‐stage dependent decline in ZFP across the stages AD, ACLF grade 1, and ACLF grade 2/3, while none were observed for PV‐1, EndoCAb IgA, LBP, or I‐FABP (Figure 1A–E).

In patients with compensated cirrhosis, circulating PV‐1 could be detected (> 0.007 ng/mL) in 2/40 (5%) of samples as compared to 89/120 (74%) samples from patients with decompensated cirrhosis (*p* < 0.0001). In addition, the percentage of patients with detectable PV‐1 increased in a stage‐dependent manner: 68% in AD (53/78), 71% in ACLF grade 1 (15/21), 100% in ACLF grade 2/3 (21/21); *p* < 0.001.

To assess correlation and proximity of the investigated biomarkers with each other as well as with markers of systemic (WBC, CRP) and hepatic inflammation (AST, ALT), portal hypertension (platelets, sodium) and liver function (bilirubin, INR, albumin, MELD), we used hierarchical cluster analysis (Figure 1F) as well as non‐parametric correlation analysis (Figure 1G). In hierarchical clustering, compensated and decompensated cirrhosis could be moderately well distinguished, primarily based on ZFP, albumin and sodium levels (Figure 1F). High proximity in hierarchical clustering across the 160 patients was observed for ZFP with albumin, PV‐1 with EndoCAb IgA, and LBP with CRP, while I‐FABP showed the least proximity to a single marker (Figure 1F).

Correlation analysis confirmed a moderate correlation of PV‐1 with EndoCAb IgA (*r*_s_ = 0.398, *p* < 0.0001) and the MELD score (*r*_s_ = 0.506, *p* < 0.0001) and its components (Figure 1G) and a weak but significant correlation with surrogates of bacterial translocation and inflammation, such as LBP (*r*_s_ = 0.179, *p* = 0.02), CRP (*r*_s_ = 0.136, *p* = 0.10) and WBC (*r*_s_ = 0.195, *p* = 0.01). Given their intercorrelation, EndoCAb IgA also correlated with MELD (*r*_s_ = 0.190, *p* = 0.02) and LBP (*r*_s_ = 0.387, *p* < 0.0001). PV‐1 and EndoCAb IgA both negatively correlated with albumin (*r*_s_ = −0.373, *p* < 0.0001 and *r*_s_ = −0.279, *p* < 0.0001, respectively), sodium (*r*_s_ = −0.222, *p* = 0.005 and *r*_s_ = −0.162, *p* = 0.04, respectively) and platelets (*r*_s_ = −0.337, *p* < 0.0001 and *r*_s_ = −0.114, *p* = 0.15, respectively).

I‐FABP levels negatively correlated with acute phase proteins (LBP: *r*_s_ = −0.273, *p* = 0.0005, CRP: *r*_s_ = −0.376, *p* < 0.0001) and positively with serum albumin (*r*_s_ = 0.232, *p* = 0.003).

ZFP concentrations negatively correlated with liver dysfunction (MELD: *r*_s_ = −0.486, *p* < 0.0001, bilirubin: *r*_s_ = −0.486, *p* < 0.0001, INR: *r*_s_ = −0.405, *p* < 0.0001) and systemic inflammation (CRP: *r*_s_ = −0.310, *p* < 0.0001, WBC: *r*_s_ = −0.239, *p* = 0.002), while the strongest positive correlation was observed with albumin (*r*_s_ = 0.444, *p* < 0.0001) (Figure 1G).

Use of non‐selective beta blockers (NSBB) was not associated.

### 3.3 Gut Permeability and Translocation Markers in the Portal Circulation

**FIGURE 2:**
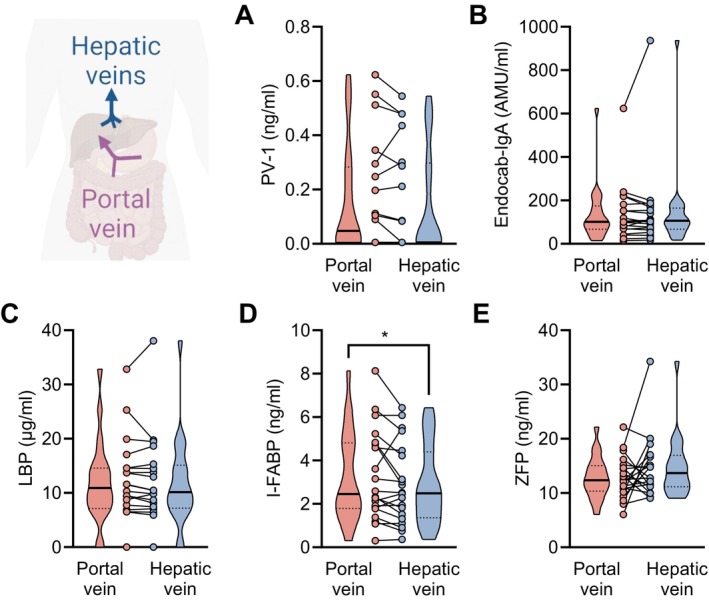
Portal vein and hepatic vein serum concentrations of surrogate markers of intestinal permeability, bacterial translocation and gut–vascular barrier in patients undergoing TIPS for recurrent/refractory ascites. Schematic representation. (A–E) Portal vein and hepatic vein serum concentrations of (A) PV‐1, (B) EndoCAb IgA, (C) LBP, (D) I‐FABP and (E) ZFP from 20 patients with decompensated cirrhosis undergoing transjugular intrahepatic portosystemic shunt (TIPS) insertion. Truncated violin plots with pairwise individual values are shown. *p < 0.05 in Wilcoxon test. EndoCAb, endotoxemia markers like endogenous endotoxin‐core antibody; I‐FABP, intestinal fatty acid binding protein; LBP, Lipopolysaccharide binding protein; PV‐1, plasmalemma vesicle protein‐1; ZFP, Zonulin‐family peptides.

We went on to investigate portal vein concentrations of the aforementioned markers in an independent cohort of 20 patients undergoing TIPS insertion for recurrent or refractory ascites (Table 2, Figure 2A). PV‐1, EndoCAb IgA, LBP and ZFP concentrations in sera derived from portal vein blood did not significantly differ from that in hepatic vein blood, while a small but significant difference in I‐FABP concentrations was observed (Figure 2B–E). Statistically, median I‐FABP concentrations were higher in the hepatic veins (median: 2.49 ng/mL) than in the portal vein (median: 2.45 ng/mL) (Figure 2D).

**TABLE 2:** Baseline characteristics of patients undergoing transjugular intrahepatic stent‐shunt (TIPS) implantation.

	Patients undergoing TIPS implantation (*n* = 20)
Age (years)	60 (56–65.75)
Male sex	9 (45%)
Aetiology	
ALD	17 (85%)
Other	3 (15%)
Child‐Pugh stage	
B	13 (65%)
C	7 (35%)
MELD score	13 (9–17)
Total serum bilirubin (mg/dL)	1.4 (0.7–2.2)
INR	1.4 (1.3–1.5)
Creatinine (mg/dL)	0.91 (0.73–1.19)
Albumin (g/L)	30 (24–32)
Sodium (mmol/L)	135 (131–139)
WBC (/nL)	8.4 (5.9–10.3)
Platelets (/nL)	158 (105–220)
ALT (U/L)	18 (15–28)
CRP (mg/L)	10.5 (8.9–16.1)

*Note:* Data are given as number with fractions or medians with interquartiles.

Abbreviations: ALD, alcohol‐related liver disease; ALT, alanine aminotransferase; CRP, C‐reactive protein; MELD, model for end‐stage liver disease; WBC, white blood cell count.

**FIGURE 3:**
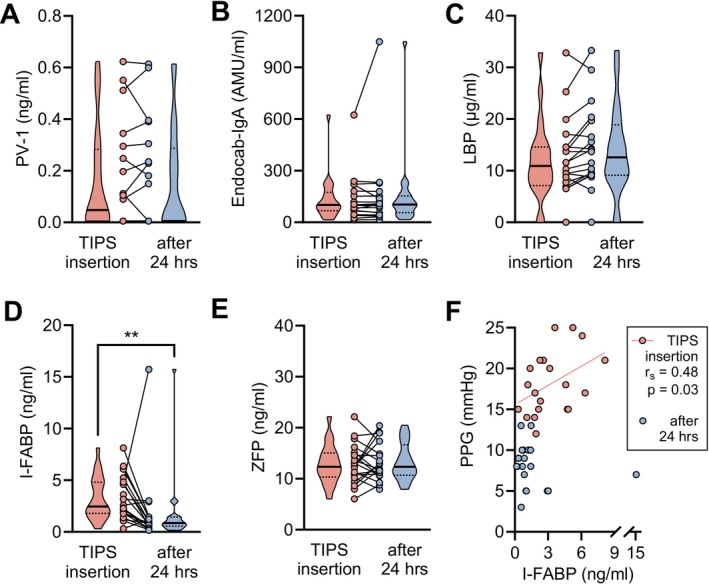
Portal vein serum concentrations of surrogate markers of intestinal permeability, bacterial translocation and gut–vascular barrier in patients undergoing TIPS for recurrent/refractory ascites and 1 day after TIPS insertion. (A–E) Portal vein serum concentrations of (A) PV‐1, (B) EndoCAb IgA, (C) LBP, (D) I‐FABP and (E) ZFP from 20 patients with decompensated cirrhosis undergoing transjugular intrahepatic portosystemic shunt (TIPS) insertion on the day of TIPS insertion and one day after. Truncated violin plots with pairwise individual values are shown. (F) Dot plot with linear regression of portal vein I‐FABP concentration and portal pressure gradient (PPG) at TIPS insertion (red) and after one day (blue). Pearson correlation coefficient with *p* value is shown. **p < 0.01 in Wilcoxon test. EndoCAb, endotoxemia markers like endogenous endotoxin‐core antibody; I‐FABP, intestinal fatty acid binding protein; LBP, Lipopolysaccharide binding protein; PPG, portal‐pressure gradient; PV‐1, plasmalemma vesicle protein‐1; ZFP, Zonulin‐family peptides.

To assess if portal decompression might change the parameters in the short term, we compared portal vein serum concentrations at TIPS insertion with concentrations sampled after 1 day. While portal vein serum concentrations of PV‐1, EndoCAb IgA, LBP and ZFP remained stable in the short term, there was a significant decrease in portal vein serum concentrations of I‐FABP at the time of TIPS insertion and the following day (median 2.45 vs. 0.85; *p* = 0.003) (Figure 3A–E). In line with that, I‐FABP portal vein serum concentrations correlated with the portosystemic pressure gradient at TIPS insertion (*r*_s_ = 0.481, *p* = 0.03) (Figure 3F). In addition, PV‐1 correlated to some degree with the PPG on the day of TIPS insertion when analysing only detectable PV‐1 samples (Figure S1).

### 3.4 The Correlation of PV‐1 With Transplant Free Survival

**FIGURE 4:**
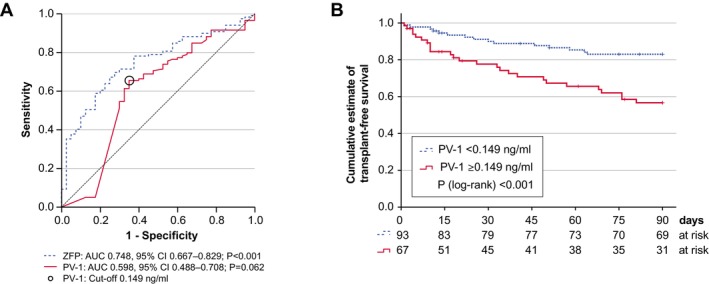
Peripheral blood serum levels of PV‐1 predict poor transplant‐free survival within 90 days after inclusion. (A) ROC curve analysis showing the diagnostic accuracy of ZFP (lower values equal higher risk) and PV‐1 (higher values equal higher risk) to predict the events death or transplant within 90 days after inclusion. Areas under the ROC curve (AUROC) and the optimum PV‐1 cut‐off are shown. (B) Kaplan–Meier analysis of transplant‐free 90‐day survival stratified for serum PV‐1 concentrations derived from (A). Patients at risk are shown. PV‐1, plasmalemma vesicle protein‐1; ZFP, Zonulin‐family peptides.

Given the association of ZFP and PV‐1 with disease stage and severity, particularly in advanced stages, we investigated the association of peripheral serum levels with outcome. Within 90 days of follow‐up, 35 patients died and six patients underwent liver transplantation. The prognostic accuracy of ZFP and PV‐1 concentrations as continuous variables for predicting the combined endpoint of death or liver transplantation within 90 days after inclusion, assessed by ROC analysis, was 0.748 (95% CI: 0.667–0.829; *p* < 0.001) and 0.598 (95% CI: 0.488–0.708; *p* = 0.062), respectively (Figure 4A). In contrast, ROC analysis revealed no significant association between 90‐day transplant‐free survival and levels of LBP (AUROC: 0.555; 95% CI: 0.444–0.666), I‐FABP (AUROC: 0.502; 95% CI: 0.392–0.613), or EndoCAb IgA (AUROC: 0.547; 95% CI: 0.440–0.655).

Since commercially available zonulin ELISA kits detect various structurally related ZFPs, whose levels decline with advancing liver disease and correlate more closely with hepatic synthetic capacity (albumin, haptoglobin) than IP in advanced cirrhosis [37, 38, 39], we focused on PV‐1 as a potentially independent prognostic biomarker for further analysis. Youden analysis revealed 0.149 ng/mL as the optimum cut‐off value for PV‐1 serum concentrations with the highest discriminative abilities. In Kaplan–Meier analysis, the cumulative estimate of transplant‐free 90‐day survival was 83.0% (standard error 4.0%) in patients with PV‐1 concentrations less than 0.149 ng/mL as compared to 56.7% (standard error 6.5%) in patients with PV‐1 concentrations of 0.149 or higher (*p* < 0.001 in log‐rank test) (Figure 4B).

In univariate Cox regression analysis, the hazard ratio of death or transplant within 90 days was 2.98 (95%: 1.58–5.63; *p* < 0.001). In multivariable Cox regression analysis, PV‐1 serum concentrations of 0.149 ng/mL or higher remained an independent predictor of death or transplant within 90 days when adjusted for age, decompensation stage, and the presence of ACLF (Table 3).

**TABLE 3:** Predictors of death or transplant within 90 days.

	Univariate Cox regression			Multivariable Cox regression		
	HR	95% CI	*p*	aHR	95% CI	*p*
PV‐1 ≥ 0.149 ng/mL	2.98	1.58–5.63	< 0.001	2.08	1.06–4.08	0.034
Age (per 1‐year increase)	1.03	1.00–1.06	0.05	1.06	1.02–1.10	0.002
Cirrhosis stage						
Never decompensated		Reference			Reference	
First decompensation	22.57	3.02–168.78	0.002	12.03	1.55–93.37	0.017
Previous decompensation	14.07	1.88–104.26	0.010	7.23	0.92–56.61	0.060
ACLF (vs. no ACLF)	4.89	2.64–9.08	< 0.001	2.09	1.55–2.81	< 0.001
Male gender (vs. female)	1.44	0.76–2.75	0.27	Removed from model^a^		
NSBB (vs. no NSBB)	1.09	0.59–2.01	0.79	Removed from model^a^		

Abbreviations: aHR, adjusted hazard ratio; ACLF, acute‐on‐chronic liver failure; CI, confidence interval; Decomp, decompensated; HR, hazard ratio; PV‐1, plasmalemma vesicle protein‐1.

^a^ Multivariable Cox regression with backward exclusion.

When liver transplantation was treated as a competing event, PV‐1 concentrations of ≥ 0.149 were significantly associated with an increased hazard of death based on Fine and Grey's proportional subhazards model (SHR: 2.15; 95% CI: 1.10–4.18; *p* = 0.02) (Figure S3).

## 4 Discussion

We herein systematically investigated the interrelationships among a well‐defined panel of proposed surrogate markers for IP, bacterial translocation and GVB damage across various stages of advanced chronic liver disease and in relation to portal hypertension. This study is the first to demonstrate that circulating PV‐1 can frequently be detected in both portal and peripheral venous blood sera from patients with decompensated cirrhosis. Increased circulating PV‐1 correlates with disease severity and is associated with a poor 90‐day transplant‐free survival rate.

Studies have challenged the traditional view that bacterial translocation in cirrhosis is confined predominantly to mesenteric lymph nodes [40]. Current research underscores a pivotal role of the GVB in promoting the translocation of bacteria directly into the portal circulation, extending to the liver and systemic pathways [41]. Increased expression of PV‐1 has been linked to increased vascular permeability, enabling the passage of macromolecules and live bacteria. This phenomenon of elevated endothelial PV‐1 expression has been documented in models of metabolic dysfunction‐associated liver disease (MASLD), cholestatic liver disease and ALD, evidencing its widespread relevance across liver pathologies [41, 42, 43, 44]. Similarly, in patients with MASLD and celiac disease, increased PV‐1 expression correlates with disease manifestations [41, 42]. Circulating PV‐1 has been proposed as a biomarker for celiac disease‐associated liver injury, illustrating its potential diagnostic utility [45].

In our cohort, PV‐1 was associated with the severity of liver disease, that is, compensation/decompensation status, albumin, platelet count, INR and bilirubin, but also with inflammatory markers such as WBC count and the acute phase proteins CRP and LBP. Further supporting a link with bacterial translocation, we observed a close clustering with endogenous endotoxin core antibodies EndoCAb IgA as a marker of cumulative endotoxin exposure [20]. The diagnostic accuracy of PV‐1 as a continuous predictor of poor 90‐day outcomes was limited by a substantial proportion of samples below the assay's detection limit; however, detectable systemic PV‐1 concentrations of 0.149 ng/mL or higher were significantly associated with poor 90‐day transplant‐free survival in univariate and multivariable Cox regression analysis and with a higher cumulative incidence of death when transplantation was treated as a competing event. PV‐1 concentrations of 0.149 ng/mL or higher remained a significant predictor of death or transplant even after adjustment for age, decompensation, or the presence of ACLF. Of note, serum PV‐1 was not significantly enriched in the portal circulation in our cohort of patients undergoing TIPS.

Given the hierarchical proximity to PV‐1, EndoCAb IgA levels were also elevated in patients with decompensated cirrhosis and correlated with liver disease severity and systemic inflammation, with the strongest correlation with PV‐1 and LBP. Previous studies have shown an increase of EndoCAb in patients with HCV infection or MASLD [46, 47], while associations with the severity of cirrhosis are scarce. In patients with PSC, higher EndoCAb IgA was associated with higher antibody levels against intracellular cytoskeletal actin filaments as a marker of structural intestinal mucosal damage [48].

The only marker among the investigated ones, which significantly correlated with the PPG in patients undergoing TIPS, was I‐FABP, an enterocyte‐specific protein, which is located in the cytosol and plays a pivotal role in the cellular uptake and metabolism of fatty acids in enterocytes [49]. In disease settings associated with intestinal mucosal damage, such as ischaemia, I‐FABP is released into circulation [24, 50] and has been implicated as a biomarker of IP [25]. Although it has been reported that the reduction of portal pressure by TIPS improves circulating I‐FABP concentrations in the longer term [51], we herein show that I‐FABP levels in portal vein serum drastically reduce within 24 h after resolution of portal hypertension. Available evidence suggests I‐FABP being a marker of portal hypertension as it correlates with the hepatovenous pressure gradient (HVPG) in patients with stable hepatitis C or alcohol‐induced cirrhosis [52], with the presence of ascites [53], and with surrogates of portal hypertension such as low platelet count [54].

Pathological IP and GVB dysfunction are early events in the progression of chronic liver disease [1]. The IP index as assessed by the lactose to mannitol excretion in urine is often pathological in patients with cirrhosis, remains rather stable in patients with mild‐to‐moderate clinically significant portal hypertension, and significantly increases with the onset of severe portal hypertension > 20 mmHg HVPG [55]. In line with that, markers of systemic inflammation, such as CPR and IL‐6, show relevant increases in patients who have already experienced decompensating events [56]. Although we have previously shown that pathological IP is accompanied by increased levels of LBP and IL‐6 in patients with cirrhosis [19], unexpectedly, we herein did not observe significantly higher LBP levels in patients with acute decompensation or ACLF. While LBP is increased in patients with ascites and hyperdynamic circulation and is of some prognostic significance in cirrhosis [57, 58], in a recent study [56] it was not significantly regulated across the stages of cirrhosis as compared to CRP, IL‐6, or procalcitonin in line with our findings. In patients with ACLF grade 2/3, we here observed even lower levels of circulating LBP despite increasing levels of CRP (Figure S2) as in acute liver failure [59]. Such stage‐dependent regulation, the high interindividual variability and its unique transcriptional regulation [21] rather argue against its use as a suitable biomarker of pathological bacterial translocation in patients with advanced liver disease.

Another biomarker with limited utility in assessing IP in patients with cirrhosis is serum zonulin [60, 61]. Zonulin which is identical to prehaptoglobin‐2 [38], is the intestinal analogue of the *Vibrio cholerae* zonula occludens toxin [62], and is involved in the regulation of the IP by decreasing the stability of tight junctions [22]. Therefore it has been proposed as a biomarker of intestinal leakage [22, 23]. Commercially available zonulin ELISA kits do not specifically target the zonulin protein alone; instead, they detect a range of structurally and functionally related proteins, collectively known as ZFPs [37]. Zonulin, the precursor of haptoglobin, is synthesised in the liver and its synthesis decreases as liver damage progresses [38, 39]. Consistent with our findings, Akpinar et al. reported that patients with decompensated cirrhosis exhibited reduced concentrations of ZFP in peripheral blood, with these levels decreasing with the stage of cirrhosis [61]. Although ZFP strongly correlated with liver disease severity and emerged as the best indicator of outcome in our cohort, its close association with serum albumin levels and inverse correlation with the MELD score and its components restrict its utility as a biomarker for IP or ‘leaky gut’ in advanced liver disease.

Our study is not without limitations. Primarily, our analysis reports intercorrelations among these serum markers within retrospective cohorts and lacks the prospective assessment of IP conducted in our previous research [19]. Previous studies, including ours, indicate that the IP index is pathologically elevated in approximately 70% of patients with compensated to almost 90% of patients with decompensated cirrhosis [19, 55]. Yet, it is uncertain whether further nominal increases in IP are associated with more severe clinical outcomes, particularly in ACLF. Secondly, we hypothesise that the PV‐1 detected in 74% of our patients with decompensated cirrhosis originates from the intestine, as proposed for celiac disease [41]. Although measurable PV‐1 was stage‐dependent, PV‐1 levels were not significantly enriched in the portal vein. Future studies are necessary to compare expression levels and serum concentrations to evaluate this hypothesis adequately. Despite speculations of hepatic origins of PV‐1 [45, 63], our data provide no evidence for significant PV‐1 accumulation in hepatic veins compared to the portal vein. Thirdly, the use of EndoCAb IgA but not IgG might be confounded by alcohol‐related aetiology of liver disease [64], which were underrepresented in compensated patient groups, and also be confounded by a defective clearance of IgA and IgA immune complexes in cirrhosis [65].

## 5 Conclusions

In summary, we present a comprehensive comparative analysis of surrogate markers of IP, bacterial translocation and GVB damage in large patient cohorts across various stages of cirrhosis, proposing circulating PV‐1 as a novel marker of gut–vascular permeability with prognostic implications. Additionally, our results validate the use of I‐FABP as a rapid biomarker for portal pressure reduction following TIPS insertion and do not support the use of serum zonulin ELISAs to assess IP in patients with chronic liver disease. Contrary to our initial hypothesis, ACLF did not correlate with an escalation in these markers. In conclusion, experimental assessment of IP by dual sugar tests remains the gold standard for evaluating gut barrier function in cirrhosis, and the efficacy of serum biomarkers must be rigorously interpreted in the context distinct stages of disease severity.

## Author Contributions

F.H. and T.B. designed the study. F.H. and J.R. performed PV‐1, EndoCAb, LBP, I‐FABP, and ZFP measurements. F.H., M.F., and T.B. performed statistical analyses. R.A., U.T., P.A.R., A.S., K.G., and T.B. collected data and organised patient recruitment. F.H. and T.B. wrote the manuscript. T.B. revised the manuscript for important intellectual content. All other authors provided intellectual input. All authors approved the final version of the manuscript.

## Ethics Statement

The study was approved by the local ethics committees (Ethikkommission der Friedrich‐Schiller Universität Jena an der Medizinischen Fakultät: 3150‐06/11, 2880‐08/10, 2018‐1080_2‐BO and Ethik‐Kommission an der Medizinischen Fakultät der RWTH Aachen: EK 327/19).

## Consent

Written informed consent was obtained from patients before study inclusion.

## Conflicts of Interest

The authors declare no conflicts of interest.

## Supporting information


Data S1.

## Data Availability

The data that support the findings of this study are available from the corresponding author upon reasonable request.
