# Supplementary material for: Surrogate Markers of Intestinal Permeability, Bacterial Translocation and Gut–Vascular Barrier Damage Across Stages of Cirrhosis
Source: Liver Int. 2025 May 2;45(6):e70119. doi: 10.1111/liv.70119 (PMC12047066; doi:10.1111/liv.70119)

Supplementary Material to the Manuscript:

Surrogate markers of intestinal permeability, bacterial translocation and gut vascular barrier damage across stages of cirrhosis

Running title:

Intestinal permeability and gut vascular barrier markers in cirrhosis

Authors:

Frederic Haedge[1], Philipp A. Reuken[2], Johanna Reißing[1], Karsten Große[1], Mick Frissen[1], Majda El-Hassani[1], Rene Aschenbach[3], Ulf Teichgräber[3], Andreas Stallmach[2], Tony Bruns[1]

Affiliations:

[1] Department of Internal Medicine III, University Hospital RWTH Aachen, Aachen, Germany.

[2] Department of Internal Medicine IV, Jena University Hospital, Friedrich Schiller University, Jena, Germany.

[3] Department of Radiology, Jena University Hospital, Friedrich Schiller University Jena, Jena, Germany.

Content:

Table S1..2

Figure S1..4

Figure S2..5

Figure S3..6

Supplementary Table S1. Types and combination of organ failures in patients with acute-on-chronic liver failure (ACLF)

**ACLF Grade 1**	n=21
Liver	5 (24%)
Kidney	11 (52%)
Brain	3 (14%)
Coagulation	2 (10%)
Circulation	0
Lungs	0
**ACLF Grade 2**	n=13
Kidney, Liver	2 (15%)
Kidney, Brain	5 (38%)
Kidney, Coagulation	2 (15%)
Liver, Brain	3 (23%)
Liver, Coagulation	1(8%)
**ACLF Grade 3**	n=8
Kidney, Liver, Coagulation, Circulation	1 (13%)
Kidney, Liver, Brain	3 (38%)
Kidney, Liver, Brain, Circulation	1 (13%)
Kidney, Brain, Circulation	1 (13%)
Liver, Brain, Coagulation	1 (13%)
Liver, Brain, Circulation	1 (13%)

Supplementary Figure S1. Correlation of portal vein serum concentration of PV-1 with portal pressure gradient (PPG) in patients undergoing TIPS for recurrent/refractory ascites

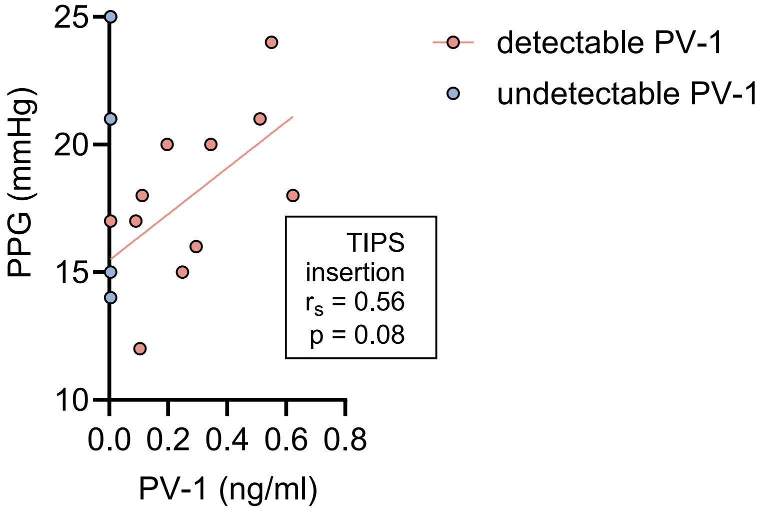

Pearson correlation coefficient with p value is shown.

Abbreviations: PV-1, plasmalemma vesicle protein-1; PPG, portal-pressure gradient

Supplementary Figure S2. Inflammatory markers across different stages of cirrhosis

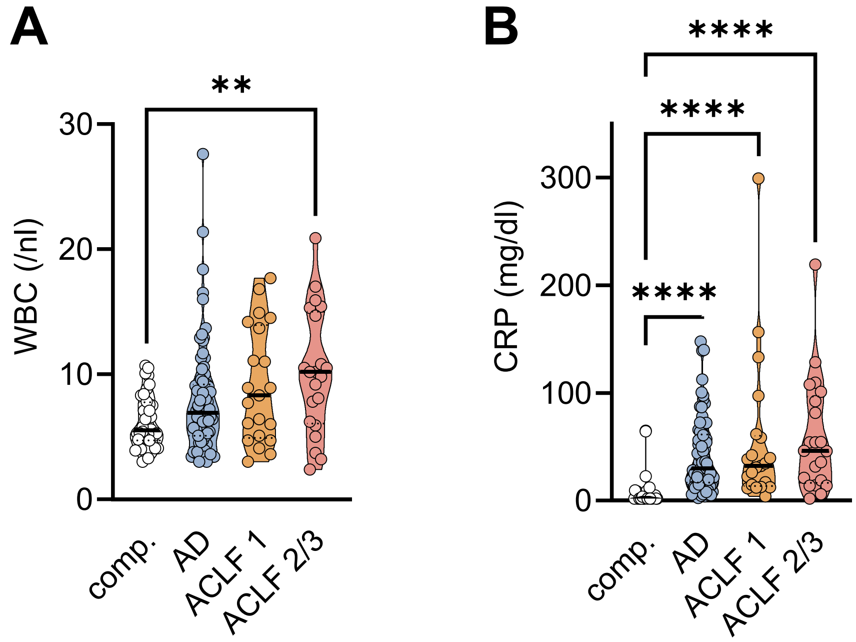

(A) White blood cell count (WBC) and (B) C-reactive protein (CRP) in patients with compensated cirrhosis (n=40), acutely decompensated cirrhosis without ACLF (AD, n=78), acute-on-chronic liver failure grade 1 (ACLF n=21) and ACLF grade 2 or higher (n=21). Truncated violin plots with overlaying individual values are shown. **p<0.01; ****p<0.0001 in Kruskal–Wallis test with Dunn's post hoc test.

Abbreviations: comp, compensated; AD, acute decompensation; ACLF, acute-on-chronic liver failure; WBC, white blood cell count; CRP, C-reactive protein

Supplementary Figure S3. Cumulative incidence function for death within 90 days treating liver transplantation as a competing event

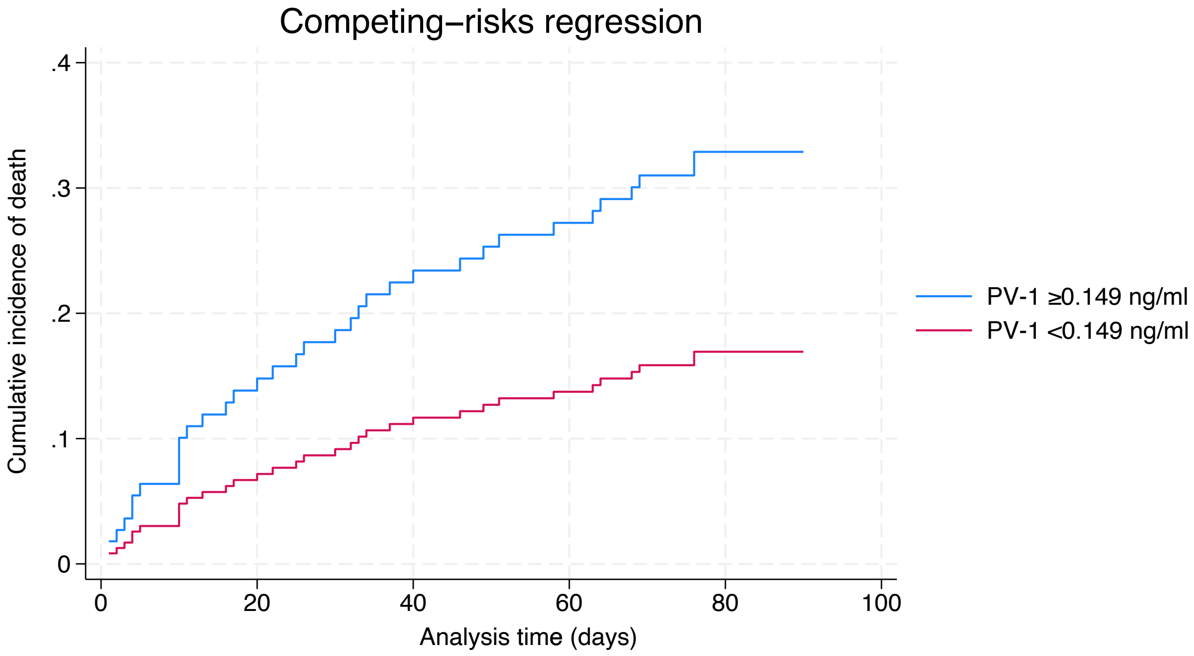

Supplement: Supplementary file 1 — Data S1. [file LIV-45-0-s001.pdf]
